# The cGAS-STING signaling pathway: emerging targets and challenges in breast cancer immunotherapy

**DOI:** 10.3389/fimmu.2025.1685931

**Published:** 2026-01-07

**Authors:** Lei Sun, Jing Chen, Shiyan Zeng

**Affiliations:** 1 Department of Radiation Oncology, Sichuan Clinical Research Center for Cancer, Sichuan Cancer Hospital and Institute, Sichuan Cancer Center, Affiliated Cancer Hospital of University of Electronic Science and Technology of China, Chengdu, China; 2 Department of Medical Oncology, Sichuan Clinical Research Center for Cancer, Sichuan Cancer Hospital and Institute, Sichuan Cancer Center, Affiliated Cancer Hospital of University of Electronic Science and Technology of China, Chengdu, China; 3 Department of Breast, Sichuan Clinical Research Center for Cancer, Sichuan Cancer Hospital and Institute, Sichuan Cancer Center, Affiliated Cancer Hospital of University of Electronic Science and Technology of China, Chengdu, China

**Keywords:** biomarker discovery, breast cancer, cGAS-STING, combination therapy, immunotherapy, immunotherapy resistance, STING agonists, tumor microenvironment (TME) remodeling

## Abstract

The cGAS-STING signaling pathway serves as a crucial bridge between innate and adaptive immunity, playing a dual role in breast cancer pathogenesis and treatment. This review delves into its complex functions within the breast tumor microenvironment, where pathway activation can either stimulate potent antitumor immunity or paradoxically promote tumor progression through immunosuppressive mechanisms. We examine the promising therapeutic strategy of utilizing STING agonists to transform immunologically quiescent tumors into T-cell-inflamed environments and their synergistic potential when combined with established modalities. The translation of these findings into clinical practice, however, faces considerable hurdles. This work critically summarizes the overarching challenges in the field and explores innovative approaches designed to overcome them. Finally, we present a forward-looking perspective on the rational development of next-generation immunotherapies centered on cGAS-STING pathway modulation, outlining key priorities for achieving its full therapeutic potential in breast cancer.

## 1 Introduction

Breast cancer, the most prevalent malignancy among women globally, continues to impose an escalating disease burden. According to the 2022 Global Cancer Statistics, approximately 2.3 million new breast cancer cases were diagnosed, accounting for 11.6% of all cancer cases (1). The International Agency for Research on Cancer (IARC) predicts this number will exceed 3 million by 2040. Although early-stage breast cancer can often achieve high cure rates through multimodal therapy, metastatic breast cancer remains a formidable therapeutic challenge, particularly due to its propensity to develop resistance to conventional treatments (2). In recent years, immunotherapy, especially immune checkpoint inhibitors (ICIs), has achieved breakthrough advances in breast cancer, offering new therapeutic options for advanced-stage patients (3). However, immunotherapy resistance remains a major obstacle limiting its clinical application.

Triple-negative breast cancer (TNBC), defined by the absence of estrogen receptor (ER), progesterone receptor (PR), and HER2 expression, has limited targeted therapy options and is associated with a poor prognosis after conventional chemotherapy (4). The advent of immunotherapy has significantly improved outcomes for a subset of TNBC patients (pembrolizumab combined with chemotherapy received FDA approval for first-line treatment of PD-L1-positive metastatic TNBC (5); the antibody-drug conjugate sacituzumab govitecan achieved a 35% objective response rate (ORR) in metastatic TNBC (6)). Despite this, TNBC immunotherapy faces significant challenges: low overall response rates and complex resistance mechanisms (4). The tumor microenvironment (TME) is a central mediator of immune resistance, involving: cancer-associated fibroblasts (CAFs) (7) and tumor-associated macrophages (TAMs) secreting immunosuppressive factors to recruit inhibitory immune cells (8); lactate accumulation due to metabolic reprogramming suppressing CD8^+^ T cell function (9); and cGAS-STING pathway dysregulation – where chromosomal instability (CIN) in TNBC can activate cGAS-STING signaling, but the induced IL-6/STAT3 axis counterintuitively promotes tumor survival (10).

Against this backdrop, targeting the cGAS-STING pathway emerges as a novel strategy to overcome immunotherapy resistance. Acting as the cell’s central alarm system for misplaced DNA, the cGAS-STING pathway is a critical sentinel that can either rally the body’s immune defenses or, if hijacked, sound a false alarm that ultimately benefits the tumor. STING agonists can remodel the TME, by activating dendritic cell (DC) antigen presentation capability, promoting CD8^+^ T cell infiltration, and reprogramming TAMs towards the anti-tumor M1 phenotype. Combination therapy (STING agonists with ICIs (11)) can reverse TME immunosuppression, with preclinical studies demonstrating significantly enhanced tumor regression rates in TNBC models. However, this pathway exhibits dual nature (inducing PD-L1 upregulation and IL-6 secretion) (12), presenting both challenges and opportunities, necessitating the development of tissue-specific delivery systems to mitigate toxicity.

To systematically analyze the translational value of the cGAS-STING pathway in breast cancer immunotherapy, this review will focus on the following key questions: 1) In-depth Mechanistic Analysis: Investigate how the cGAS-STING pathway regulates the CAF-TAM-MDSC interaction network and metabolic reprogramming within the breast cancer TME. 2) Strategies to Overcome Resistance: Evaluate the potential of novel STING agonists (oral MSA-2, nanocarrier delivery systems) to overcome ICI resistance. 3) Bottlenecks in Clinical Translation: Analyze solutions to the pathway’s dual effects (pro-metastatic inflammatory responses) and propose precise intervention strategies. By integrating the latest basic research and clinical progress, we aim to provide new targeted approaches and combination therapy paradigms for breast cancer immunotherapy.

## 2 Immunotherapy breakthroughs and biomarker evolution in breast cancer

### 2.1 TNBC: from metastatic to early-stage paradigm shifts

In recent years, immunotherapy for TNBC has achieved landmark progress (13). In first-line treatment for advanced disease, PD-1/PD-L1 inhibitors combined with chemotherapy have become the standard therapy for PD-L1-positive (Combined Positive Score [CPS] ≥10) metastatic TNBC (14). Based on data from the KEYNOTE-355 trial (15), pembrolizumab combined with chemotherapy significantly prolonged progression-free survival (PFS; hazard ratio [HR] = 0.65), leading to its FDA approval for patients with CPS ≥10. While atezolizumab combined with nab-paclitaxel (IMpassion130 (16)) demonstrated OS benefit in the PD-L1-positive (SP142 assay) population, its confirmatory trial, IMpassion131, failed to meet its primary endpoint, highlighting the challenge of biomarker assay platform consistency (17).

The rise of antibody-drug conjugates (ADCs) has further expanded the therapeutic landscape. *Sacituzumab govitecan* (SG) monotherapy achieved an objective response rate (ORR) of 31% in the ASCENT trial (18). A phase II trial (NCT03547973 (19)) evaluating SG combined with pembrolizumab demonstrated synergistic potential. Notably, a novel TROP2-targeted ADC (20), datopotamab deruxtecan (Dato-DXd) demonstrated promising clinical activity (ORR = 26.8% in HR+/HER2- BC; ORR = 31.8% in TNBC) and a manageable safety profile (stomatitis being the most common adverse event) in heavily pretreated patients with advanced HR+/HER2- breast cancer or triple-negative breast cancer, and is currently under evaluation in phase III studies (21).

In the early-stage TNBC setting, neoadjuvant immunotherapy has yielded transformative advances. The KEYNOTE-522 trial confirmed that pembrolizumab combined with chemotherapy significantly increased the pathological complete response (pCR) rate (absolute increase of 13.6 percentage points; 64.8% *vs*. 51.2%) (22), crucially, this benefit was observed independently of PD-L1 status. This regimen has been established as the new standard of care for high-risk early-stage TNBC and has been extended into the adjuvant setting. In the phase 3 KEYNOTE-119 trial, pembrolizumab monotherapy did not significantly improve overall survival compared to single-agent chemotherapy in patients with previously treated metastatic triple-negative breast cancer, including in subgroups defined by PD-L1 expression (CPS ≥1 or CPS ≥10), despite a numerical trend favoring pembrolizumab in the CPS ≥10 subgroup (HR 0.78, p=0.057) (23).

### 2.2 Extending immunotherapy to HR+ and HER2+ subtypes

Immunotherapy for hormone receptor-positive (HR+) breast cancer remains challenging, primarily due to its low immunogenicity and an immunosuppressive tumor microenvironment (TME) mediated by regulatory T cells (Tregs) and CAFs. To overcome this hurdle, targeted-immunotherapy combinations are a major research focus: The combination of the CDK4/6 inhibitor palbociclib with pembrolizumab demonstrated clinical efficacy in in patients with hormone receptor-positive metastatic breast cancer (NCT02778685 (24)). Moreover, the combination of the PARP inhibitor olaparib with durvalumab showed synergistic anti-tumor activity in patients (25). Immunotherapy exploration in HER2-positive (HER2+) breast cancer is centered on ADC-ICI combinations (26). The novel ADC trastuzumab deruxtecan (T-DXd) combined with ICIs also demonstrates therapeutic potential for the HER2-positive subtype (27).

### 2.3 The evolving landscape of predictive biomarkers

Current biomarker research is evolving from single indicators towards multidimensional integration. PD-L1 expression, a traditional predictive biomarker, is limited by assay platform variability (low concordance between SP142 and 22C3 antibodies) (28). The benefit of neoadjuvant immunotherapy in PD-L1-negative patients in KEYNOTE-522 (29) exemplifies its limitations. Significantly, PD-L1 expressed by CAFs and tumor-associated macrophages (TAMs) also contributes to adaptive immune resistance, highlighting the immunomodulatory role of non-malignant cells within the TME.

Higher tumor-infiltrating lymphocyte (TIL) abundance is significantly associated with improved survival outcomes in patients with early-stage triple-negative breast cancer who did not receive chemotherapy (30). Single-cell sequencing further revealed that enrichment of CXCL13+ T cells within the TME positively correlates with response to anti-PD-L1 therapy (31), underscoring the predictive value of immune cell spatial heterogeneity. Novel strategies advocate integrating neoantigen quality, T cell clonality, and metabolic features (Lactate drives TNBC immunosuppression via CXCL12/CXCR4 and enables lactate-based prognostics for immunotherapy (32)) to build comprehensive predictive models.

Immune Gene Signatures: Activation status of the cGAS-STING pathway can predict response to STING agonists. A single-cell spatial transcriptomics-identified FOLR2+ macrophage subset (33), positively correlated with CD8+ T cell infiltration, indicates favorable prognosis. Epigenetic Regulation: EZH2 overexpression causes T cell exclusion via epigenetic silencing mechanisms, and its inhibitor combined with ICIs can reverse immune resistance (34).

Substantial progress elucidates CAF-mediated resistance mechanisms: FAP+ CAFs exclude CD8+ T cells, while targeting circNOX4 can reprogram CAFs to enhance T cell infiltration (35). TAM spatial heterogeneity is also being unraveled – a dynamic balance between pro-tumor CX3CR1+ TAMs (36) within the TME constitutes a novel therapeutic target. Furthermore, metabolic reprogramming (high LDHA expression) is confirmed to shape an immunosuppressive microenvironment, and combining metabolic pathway targeting with ICIs exhibits synergistic potential.

## 3 Navigating challenges and future directions in TNBC immunotherapy

Although immunotherapies, particularly pembrolizumab-based regimens, have reshaped the TNBC treatment landscape, a critical examination of their clinical limitations is essential to guide future progress.

### 3.1 Enduring hurdles: efficacy, biomarkers, and resistance

Despite significantly improved progression-free survival (PFS) with pembrolizumab-chemotherapy in PD-L1-positive (CPS ≥10) previously untreated locally recurrent inoperable or metastatic triple-negative breast cancer (median PFS 9.7 *vs*. 5.6 months; HR 0.65), deep responses remain limited—highlighting persistent therapeutic challenges (15).

Despite a clinically meaningful overall survival (OS) improvement with atezolizumab plus nab-paclitaxel in PD-L1 IC-positive metastatic TNBC (median OS 25.4 *vs*. 17.9 months; HR 0.67), the absence of long-term survival plateaus underscores the impact of primary or acquired resistance in most patients (37).

In KEYNOTE-522’s exploratory analysis, pembrolizumab plus neoadjuvant chemotherapy followed by adjuvant pembrolizumab significantly increased pathological complete response (pCR/RCB-0) rates, shifted residual cancer burden (RCB) distributions toward lower categories, and improved event-free survival (EFS) across all RCB subgroups—with the greatest benefit in RCB-2 patients (HR = 0.52)—demonstrating efficacy even in non-pCR populations (29).

**Figure 1:**
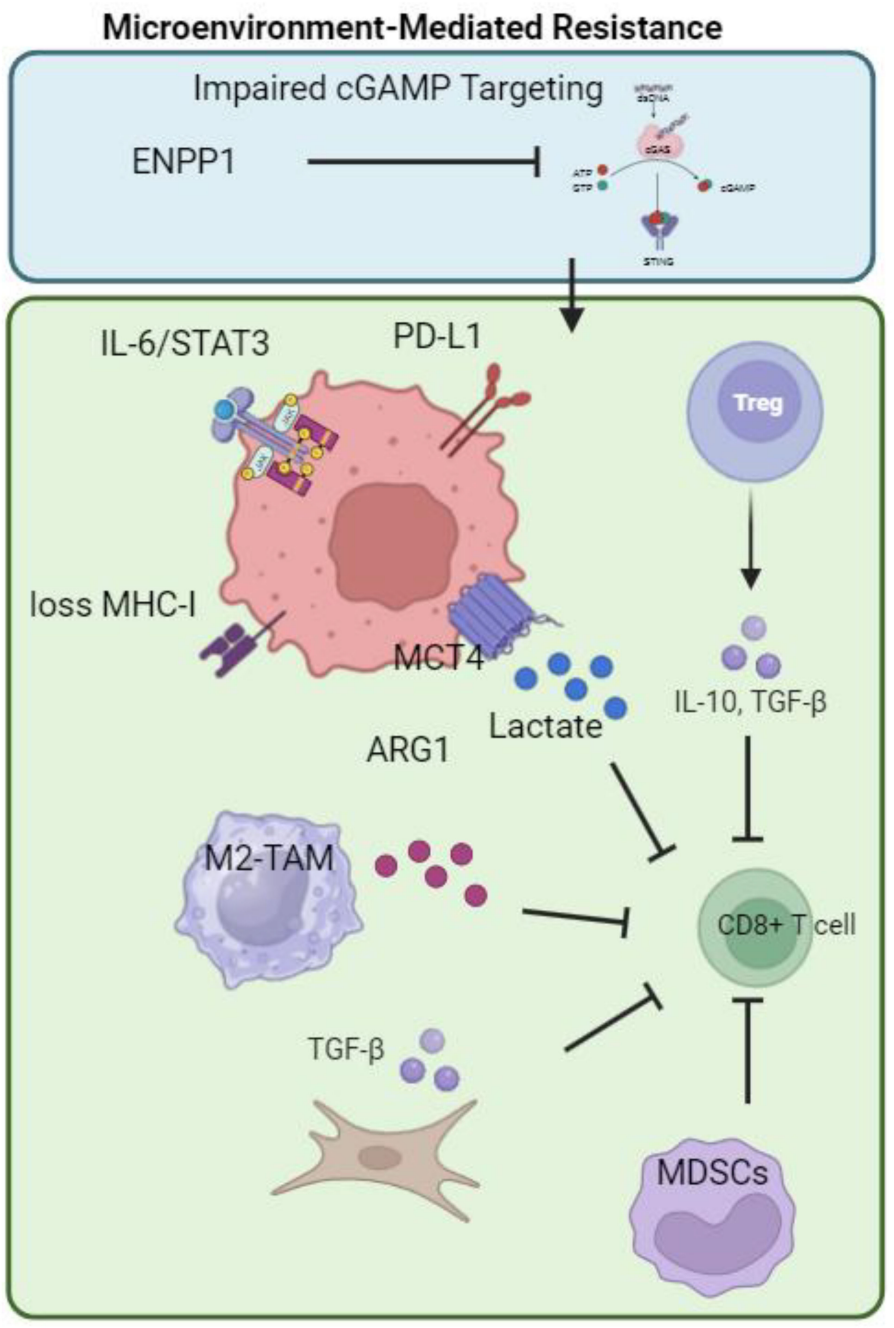
Mechanisms of immunotherapy resistance in TNBC. This model illustrates the key mechanisms limiting immunotherapy efficacy in triple-negative breast cancer (TNBC). Tumor cell-intrinsic adaptations include PD-L1 upregulation, IL-6/STAT3 survival signaling, and MHC-I loss. The immunosuppressive tumor microenvironment features Tregs, MDSCs, and M2-TAMs that inhibit CD8^+^ T cell function, alongside CAFs that promote T-cell exclusion through ECM remodeling. Metabolic immunosuppression via lactate accumulation and enzymatic degradation of cGAMP by ENPP1 further dampen antitumor immunity. Created by BioRender.com (YQ294GQM5T).

Unreliable PD-L1 Predictive Value: The utility of PD-L1 as a biomarker is hampered by significant spatiotemporal heterogeneity, inconsistent detection methodologies (CPS *vs*. IC scores; variability between 22C3 and SP142 assays (38)), and reduced predictive power in the neoadjuvant compared to the metastatic setting, as observed in the GeparNuevo trial (39). As illustrated in **Figure 1**, the mechanisms underlying immunotherapy resistance in TNBC are complex, involving multiple factors such as intrinsic tumor cell adaptive immune evasion, an immunosuppressive tumor microenvironment, metabolic reprogramming, and cGAMP degradation.

Tumor Mutational Burden (TMB): Only ~20% of TNBCs exhibit TMB-high (≥10 mut/Mb) (40), and its correlation with ICI response is notably weaker than in lung cancer. Immune Gene Signatures: Profiles like interferon-gamma (IFN-γ) signaling (41) and the T-cell inflammatory gene expression profile (TIS) show promise in clinical trials but need broader validation (42). Liquid Biopsy: Prospective ctDNA surveillance identifies early-stage TNBC patients at high relapse risk, yet pembrolizumab failed to achieve sustained ctDNA clearance in detected cases—highlighting challenges in intercepting molecular relapse (43).

While TNBC was historically classified among immunologically ‘cold’ tumors, it has emerged as the breast cancer subtype most responsive to immune checkpoint inhibitors—with pembrolizumab now approved in both neoadjuvant and advanced settings (44). In TNBC, M2-polarized tumor-associated macrophages (TAMs; CD163+) suppress effector T-cell function via ARG1, while exosomal LAP-TGF-β1 contributes to immunosuppression by reshaping the metastatic niche (45, 46). Metabolic remodeling in TNBC leads to lactate accumulation (via high MCT4 expression), which promotes PD-L1-mediated immunosuppression and may indirectly impair T-cell function (47).

In metastatic breast cancer, β2-microglobulin (B2M) mutations emerge as a positive correlate of T-cell inflamed signatures specifically within TMB-high tumors—highlighting their potential role in composite biomarkers for immune checkpoint inhibitor response (48). Oncogenic Pathway Activation: In TNBC, PI3K/AKT/mTOR pathway hyperactivation promotes metastasis and may indirectly impair T-cell infiltration by enhancing immunosuppressive factors (49). Host Factors: Gut microbiome dysbiosis (loss of beneficial taxa like Ruminococcus torques (50) and systemic inflammation (reflected by NLR/PLR) is associated with ICI efficacy, though its role in irAEs requires further investigation (51).

During neoadjuvant treatment, the incidence of grade 3 or greater immune-related adverse events with immune checkpoint inhibitors (ICIs) was 10.3% across early breast cancer patients receiving combination therapy (2). In the DOP arm (durvalumab/olaparib/paclitaxel), 12.3% of patients with HER2-negative breast cancer experienced immune-related grade 3 adverse events (25) (**Table 1**).

**Table 1:** Key therapeutic strategies in TNBC.

Therapeutic strategy	Representative examples	Development status
Immune Checkpoint Inhibitors	Pembrolizumab, Atezolizumab	Approved
Antibody-Drug Conjugates (ADCs)	Sacituzumab Govitecan, Datopotamab Deruxtecan	Approved/Phase III
PARP Inhibitors	Olaparib	Approved (gBRCAm)
STING Agonists	MK-1454, MSA-2	Phase I/Preclinical
Cell Therapy	ROR1 CAR-T	Phase I/Preclinical
Novel Immunotherapy Combinations	Entinostat + Pembrolizumab, Galunisertib + Durvalumab	Phase I/II

The substantial intertumoral heterogeneity of TNBC critically impacts response to immunotherapy. Cancer-associated fibroblasts (CAFs) in the tumor microenvironment (TME), which exhibit signaling including transforming growth factor-beta (TGF-β), are critical for promoting therapeutic resistance (52). The Luminal Androgen Receptor (LAR) subtype of TNBC is characterized by a high rate of PIK3CA mutation and deletions in CD274 (PD-L1) and PDCD1LG2 (PD-L2), along with a low homologous recombination deficiency (HRD) score (53) (**Table 2**). TNBC exhibits highly heterogeneous responses to immune checkpoint blockade (ICB), where high tumor mutational burden (TMB) correlates with exceptional efficacy, while PD-L1 and tumor-infiltrating lymphocytes (TILs) showed no significant predictive value; regulatory T-cell markers were enriched in responders, whereas GARP overexpression was associated with rapid progression and poor prognosis (54).

**Table 2:** Combination therapeutic strategies targeting cGAS-STING in preclinical TNBC models.

Strategy	Mechanistic basis	Trial design/status
STING agonist + ICI	Nanoparticle-enabled tumor-restricted STING activation transforms immunosuppressive microenvironment	The MSA-2-activatable nanoadjuvant synergizes with ICIs against tumors (103)
STING agonist + PARPi	Synergistic DNA damage (BRCA-mutated TNBC); PARPi-induced cytosolic DNA amplifies STING signaling	Preclinical TNBC models (diABZI + PARPi) in BRCA-mutated TNBC model (93)
Novel Delivery	Nanoparticles (cGAMP-NP (104)) enhance tumor-selective cGAMP delivery	In preclinical TNBC models, cGAMP-NPs suppress tumors by activating STING, reprogramming macrophages, and overcoming PD-L1 resistance

### 3.2 The path forward: novel combinations and precision strategies

Building robust predictive algorithms for platinum-based neoadjuvant chemotherapy response in TNBC will require further investigation of genomic features, particularly homologous recombination deficiency (HRD) status (55). Spatial proteomic profiling (43-plex IMC) of serial biopsies from a neoadjuvant ICB trial in TNBC revealed that response is driven by multicellular spatial organization—specifically pre-treatment proliferating CD8+TCF1+ T cells and MHCII+ cancer cells, alongside on-treatment expansion of granzyme B+ T cells in responders versus CD15+ cancer cells in non-responders—demonstrating that early on-treatment tissue features combined with baseline signatures optimally predict response (56). A pretreatment perfusion MRI-based radiomics score, derived from pharmacokinetic parameters (Ktrans, Ve, Slopemax), demonstrated good predictive value for pathologic complete response (pCR) to neoadjuvant chemoimmunotherapy in early-stage triple-negative breast cancer (AUC = 0.80), and a nomogram combining this score with grade and Ki-67 further improved prediction (AUC = 0.86), showing correlation with tumor immune environment features (57). Novel Combination Strategies Targeting Resistance Mechanisms: Future therapeutic advancements hinge on rationally designed combinations (**Table 3**).

**Table 3:** Investigational combination strategies to overcome immunotherapy resistance.

Combination strategy	Representative agents/trials	Mechanism/rationale
Targeting Immunosuppressive Pathways	ICI + TGF-βR inhibitor (Galunisertib; NCT02734160 (58))	Reverses Treg-mediated suppression and remodels the ECM
Activating Innate Immunity	ICI + STING agonist (ulevostinag; NCT03010176 (59))	Promotes dendritic cell maturation and T-cell priming
Metabolic Modulation	ICI + IDO1 inhibitor (Epacadostat (60))	Counteracts T-cell suppression mediated by tryptophan depletion
ADC/Bispecific Antibody + ICI	ICI + ADC (TROP2 (61))	TROP2 controls immune exclusion, enhances immunotherapy
Epigenetic Modulation + ICI	ICI + HDAC inhibitor (Entinostat; NCT02697630 (62))	Enhances anti-tumor immune responses

Biomarker-Driven Treatment Selection: Patient stratification based on molecular profiling enables tailored regimens: 1) PD-L1-positive: Frontline ICI-chemotherapy. 2) HRD-positive/mBRCAm: ICI + PARPi maintenance therapy. 3) LAR subtype: Exploration of ICI + androgen receptor inhibitors (Enzalutamide (63)). Refining Therapeutic Sequencing: Prioritizing ICI use in the neoadjuvant phase has demonstrated significant benefit (KEYNOTE-522 (29)), potentially maximizing T-cell priming. In the adjuvant phase, personalized maintenance strategies guided by biomarkers (intensified ICI for patients with persistent ctDNA positivity) are crucial for preventing relapse.

Bispecific Antibodies: Agents like PD-1 and TIGIT bispecific antibodies (64) offer simultaneous blockade of multiple immune evasion pathways. Cell-Based Therapies: Chimeric antigen receptor (CAR) T-cells targeting antigens like ROR1 (65) or off-the-shelf approaches like allogeneic CAR-T cells are in development (66). Microbiome Modulation: Interventions such as specific probiotics [Akkermansia muciniphila (67)] or fecal microbiota transplantation (FMT) (68) are being investigated to enhance ICI efficacy by modulating the gut microbiome.

## 4 The cGAS-STING signaling pathway: core mechanisms and functions

The cGAS-STING pathway serves as a critical bridge between innate and adaptive immunity, enabling the host to detect cytoplasmic DNA and trigger robust inflammatory responses (69). This review systematically examines the role of cGAS-STING in shaping the breast cancer immune microenvironment, its therapeutic targeting, clinical translation status, and future research directions.

### 4.1 Molecular components and activation

The Initial Alarm: DNA Sensing and cGAMP Production: cGAS functions as a cytosolic DNA sensor that recognizes aberrant double-stranded DNA (dsDNA) derived from tumor-associated sources, including micronuclear DNA, mitochondrial DNA (mtDNA), neutrophil extracellular traps (NETs), and exosomal DNA. Upon ligand binding, cGAS catalyzes the synthesis of the cyclic dinucleotide 2’,3’-cyclic GMP-AMP (cGAMP) from ATP and GTP, serving as a secondary messenger.

Relaying the Signal: STING Activation and Trafficking: STING, an endoplasmic reticulum (ER)-resident transmembrane protein, acts as the direct receptor for cGAMP. cGAMP binding induces conformational changes in STING (70), facilitating its trafficking from the ER to the Golgi apparatus—a prerequisite for downstream signaling initiation.

### 4.2 Downstream signaling cascades

**Figure 2:**
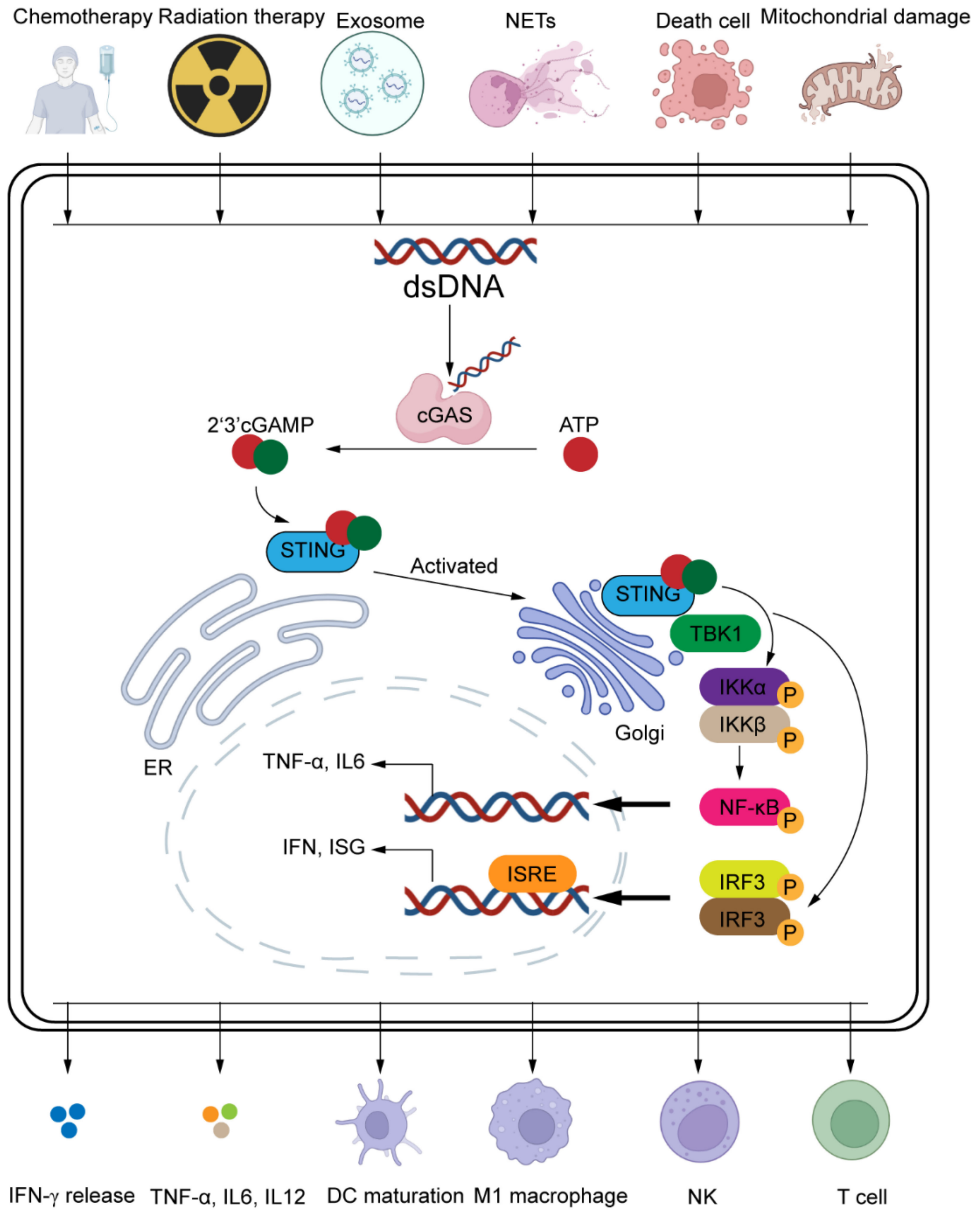
Molecular mechanism of the cGAS-STING signaling pathway. Upon cytoplasmic double-stranded DNA (dsDNA) exposure (from micronuclei, mitochondrial release, or therapy-induced damage), cGAS binds dsDNA and synthesizes the cyclic dinucleotide 2’3’-cGAMP. cGAMP activates STING on the endoplasmic reticulum (ER), triggering its translocation to the Golgi. STING recruits TBK1, which phosphorylates IRF3 and IKK (activating NF-κB). Phosphorylated IRF3 dimerizes and translocates to the nucleus to induce type I interferons (IFN-α/β) and interferon-stimulated genes (ISGs) via ISRE elements. NF-κB drives transcription of pro-inflammatory cytokines (TNF-α, IL-6). Key outputs include dendritic cell (DC) maturation, M1 macrophage polarization, and activation of NK/CD8+T cells. Visual: Color-coded molecules; arrows indicate cascade progression. ER, endoplasmic reticulum. Created by BioRender.com (YQ294GQM5T).

The Immune Call to Arms: IRF3 and NF-κB Activation: Following cGAMP binding, STING traffics from the ER to the Golgi apparatus and recruits TANK-binding kinase 1 (TBK1), which phosphorylates interferon regulatory factor 3 (IRF3). Phosphorylated IRF3 undergoes dimerization and nuclear translocation, driving the expression of type I interferons (IFN-α/β) and interferon-stimulated genes (ISGs). Concurrently, STING activates the NF-κB pathway, leading to pro-inflammatory cytokines production (71). This dual signaling arms synergistically amplify immune activation and antigen presentation. As illustrated in **Figure 2**, this pathway senses cytosolic dsDNA via cGAS and synthesizes cGAMP, which subsequently activates the STING-TBK1-IRF3/NF-κB axis to induce the expression of type I interferons and pro-inflammatory cytokines, thereby initiating antitumor immunity.

### 4.3 Core biological functions: orchestrating antitumor immunity

The cGAS-STING pathway integrates innate and adaptive immune responses, serving as a pivotal “command center” for anti-tumor immunity. When the cytosolic DNA sensor cGAS recognizes tumor-derived double-stranded DNA (such as micronuclear DNA, mitochondrial DNA, or exosomal DNA), it catalyzes the synthesis of the second messenger 2’,3’-cGAMP. This messenger binds STING, which is primarily localized to the endoplasmic reticulum (ER) (72). Subsequently, the activated STING translocates via the ER-Golgi intermediate compartment (ERGIC) to the Golgi apparatus, where it recruits TBK1. TBK1 directly phosphorylates and activates IRF3. Simultaneously, the STING-TBK1 signaling pathway activates the IKK complex (leading to IκBα degradation) or recruits adaptor proteins like TRAF6, ultimately leading to the release and activation of the NF-κB transcription factor (73). These activated transcription factors collectively drive the expression of type I interferons and various pro-inflammatory cytokines, initiating a local and systemic immune activation cascade. Critically, this signaling:

Enhances antigen presentation by promoting dendritic cell (DC) maturation and cross-priming of tumor antigens to CD8^+^ T cells (74); Activates cytotoxic effector cells via recruitment and functional augmentation of natural killer (NK) cells and tumor-specific CD8^+^ T lymphocytes; Reprograms immunosuppressive networks by polarizing tumor-associated macrophages (TAMs) toward pro-inflammatory M1 phenotypes while inhibiting regulatory T cells (Tregs) and myeloid-derived suppressor cells (MDSCs); Counters metabolic immunosuppression through downregulation of lactate dehydrogenase A (LDHA), thereby mitigating lactate-mediated T cell dysfunction. Collectively, these mechanisms transform “cold” tumors into immunologically “hot” microenvironments, enabling sustained antitumor surveillance and tumor cell clearance.

cGAS-STING activation influences cellular senescence in a context-dependent manner. Sustained signaling induces senescence-associated secretory phenotypes (SASPs) (75), while acute activation can trigger tumor cell death.

### 4.4 miRNA-mediated regulation of cGAS-STING

MicroRNAs (miRNAs) serve as critical upstream fine-tuners of the cGAS-STING pathway, maintaining immune homeostasis in physiology and contributing to dysregulation in pathology. Physiological Function: Under homeostatic conditions, specific miRNAs prevent excessive and potentially harmful inflammatory responses by targeting core components of the pathway. For instance, circTMEM56 was found to function as a molecular sponge for miR-136-5p, thereby upregulating STING expression and enhancing cGAS-STING-dependent interferon signaling (76). In TNBC, upregulation of miR-181a directly suppresses STING expression, dampening the interferon response and driving resistance to PARP inhibitors and platinum-based chemotherapy (77).

## 5 Research advances in cGAS-STING pathway for cancer immunotherapy

### 5.1 cGAS-STING as an endogenous immune activation hub

The cGAS-STING pathway serves as a pivotal immune surveillance mechanism activated by cytoplasmic double-stranded DNA resulting from ‘leaky’ DNA end resection following 53BP1 loss in BRCA1-deficient cells (78). DNA-damaging modalities—including radiotherapy, chemotherapy, and targeted agents (PARP inhibitors, topoisomerase inhibitors)—induce cytosolic dsDNA accumulation, triggering cGAS-dependent cGAMP synthesis and STING activation. This cascade promotes type I interferon (IFN-I) secretion and pro-inflammatory cytokine production, priming adaptive immunity. Notably, ionizing radiation stimulates cGAS-STING-CCL5 signaling (79), while PARP inhibitors synergize with CAR-T cells by activating cGAS-STING in tumor cells (80).

Intrinsic genomic instability—driven by telomere attrition, homologous recombination deficiency (HRD), or spindle assembly checkpoint defects—generates micronuclei that release dsDNA into the cytosol (81). Chronic cGAS-STING activation in this context induces a senescence-associated secretory phenotype (SASP), fostering a pro-metastatic microenvironment. Notably, in TNBC, chromosomal instability (CIN)-driven STING signaling sustains tumor survival via IL-6/STAT3 activation (10).

### 5.2 Therapeutic targeting: STING agonists and delivery platforms

STING agonist development has progressed through three classes: natural cyclic dinucleotides (CDNs) such as c-diGMP, c-diAMP, and 2′3′-cGAMP enhance dendritic cell maturation and T-cell responses but exhibit poor pharmacokinetics (82). Synthetic CDNs (ADU-S100 (83), MK-1454) feature phosphorothioate modifications improving stability. ADU-S100 showed limited monotherapy efficacy in phase I solid tumor trials (NCT02675439) (84), while MK-1454 combined with pembrolizumab induced regression in non-injected lesions. Non-nucleotide small molecules (diABZI, MSA-2) enable oral or systemic administration. The PEIM nanoadjuvant co-delivering MSA-2 synergized with immune checkpoint blockade therapy to prevent postoperative tumor recurrence and distant metastasis in MC38 colorectal and B16-OVA melanoma models (85). Delivery challenges persist due to cGAMP’s hydrophilicity and enzymatic degradation by ENPP1 (86).

Combination strategies robustly enhance efficacy: Immune checkpoint inhibitors (ICIs): STING agonists increase tumor-infiltrating CD8+ T cells and reverse ICI resistance. Radiotherapy/DNA-damaging agents: Amplify immunogenic cell death and cytosolic DNA release. Tumor vaccines: STINGVAX (87) suppressed melanoma growth by enhancing DC and T-cell infiltration.

**Figure 3:**
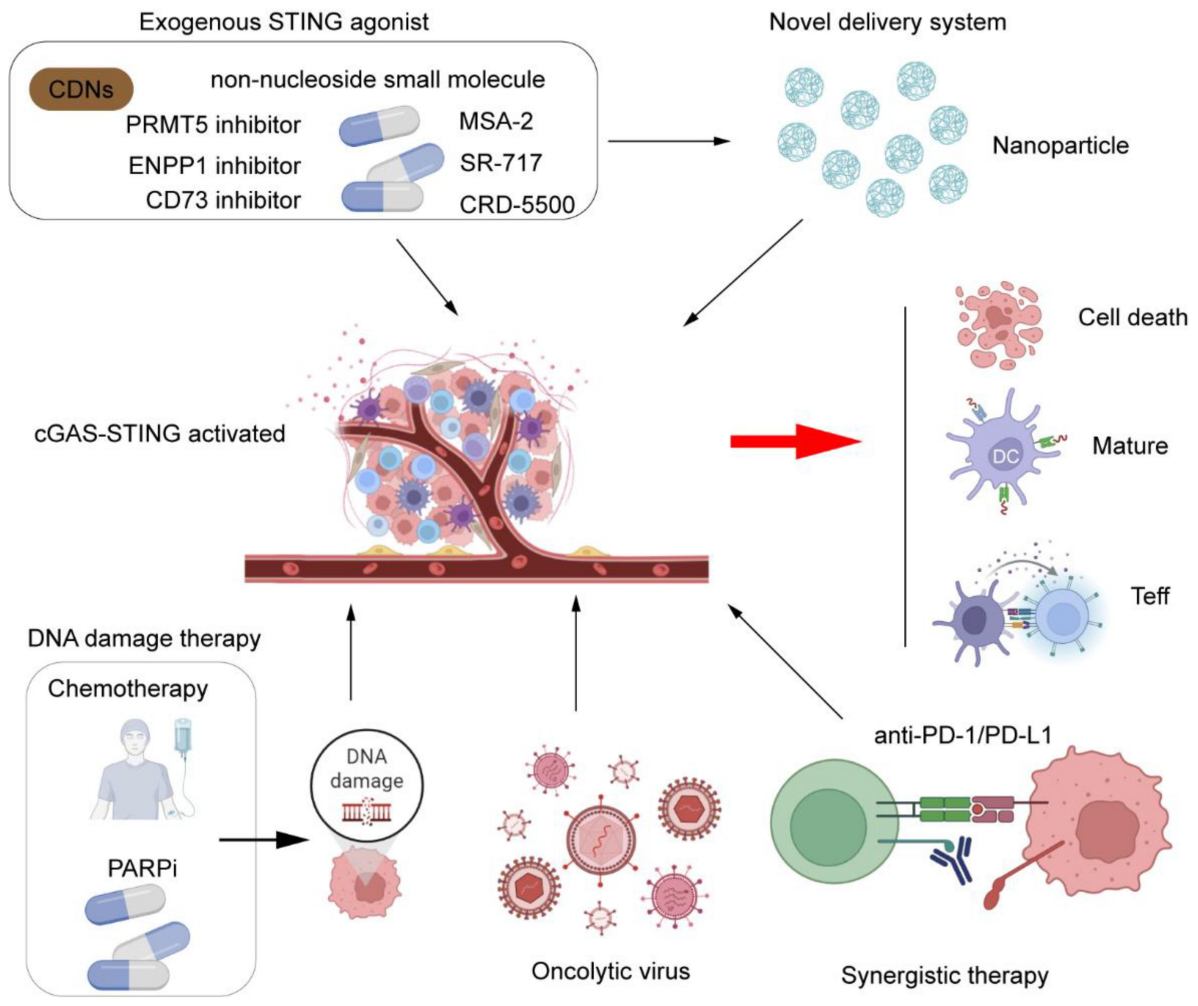
Therapeutic strategies targeting cGAS-STING in breast cancer. Direct activation: STING agonists (cyclic dinucleotides/non-nucleotide compounds) are delivered intravenously or intratumorally, optionally via nanocarriers for targeted immune cell delivery. Combination approaches: DNA-damaging agents (radiotherapy, chemotherapy, PARPi) induce tumor dsDNA, initiating endogenous cGAS-STING signaling. Immune checkpoint inhibitors (anti-PD-1/PD-L1) synergize with STING agonists to reverse T-cell exhaustion. Effects: Agonists enhance DC maturation/lymph node migration, activating naïve T cells into tumor-infiltrating lymphocytes (TILs). Coordinated CTL/NK/M1 macrophage activity induces tumor cell death. Visual: Color-coded treatment modalities; lightning symbols denote STING activation; dashed arrows indicate cell migration. Created by BioRender.com (YQ294GQM5T).

Beyond conventional STING agonists, novel strategies are advancing to activate the pathway. The oncolytic virus T-VEC induces cGAS-STING pathway activation through viral DNA release, with the SOLTI-1503 PROMETEO trial investigating its combination with atezolizumab (anti-PD-L1) to achieve residual cancer burden 0/1 in operable HER2-negative breast cancer patients presenting residual disease after neoadjuvant chemotherapy (88). Nanoparticle delivery systems enhance tumor-specific agonist delivery while minimizing systemic toxicity, showing promising efficacy in preclinical TNBC models. Additionally, epigenetic modulators such as EZH2 and HDAC inhibitors can reverse the silencing of the IFI16-STING-CXCL10/11 signaling pathway in HER2+ breast cancer, priming tumors for trastuzumab response and creating novel combinatorial therapeutic opportunities for clinical translation (89). As shown in **Figure 3**, current therapeutic strategies targeting the cGAS-STING pathway include STING agonists, nanocarrier delivery systems, and their combination with radiotherapy, immune checkpoint inhibitors (ICIs), and PARP inhibitors, aiming to enhance the antitumor immune response.

### 5.3 Clinical translation challenges

Despite preclinical promise, clinical application faces hurdles: Limited single-agent activity: ADU-S100 monotherapy showed marginal efficacy in advanced cancers. Tumor-intrinsic suppression: ENPP1 overexpression in breast cancer generates adenosinergic metabolites that upregulate haptoglobin (HP), promoting polymorphonuclear myeloid-derived suppressor cell (PMN-MDSC) infiltration and neutrophil extracellular trap (NET) formation, ultimately facilitating locoregional relapse post-surgery/irradiation. This immune remodeling parallels mechanisms where ENPP1 overexpression and MDSCs dampen immune activation (90). Pro-tumor effects: Chronic STING signaling induces PD-L1/IDO upregulation and NF-κB-driven IL-6 production. Brain metastasis is facilitated by cGAMP transfer from tumor cells to astrocytes, activating STAT1/NF-κB.

## 6 cGAS-STING pathway in breast cancer: mechanisms and therapeutic implications

### 6.1 Subtype-specific pathway activation

Breast cancer subtypes exhibit divergent regulation of the cGAS-STING pathway. TNBC demonstrates chromosomal instability-driven activation, with cytosolic DNA inducing IL-6 production. By contrast, basal-like and luminal subtypes may silence the pathway via epigenetic mechanisms akin to melanoma.

### 6.2 Double-edged sword in tumor microenvironment

**Figure 4:**
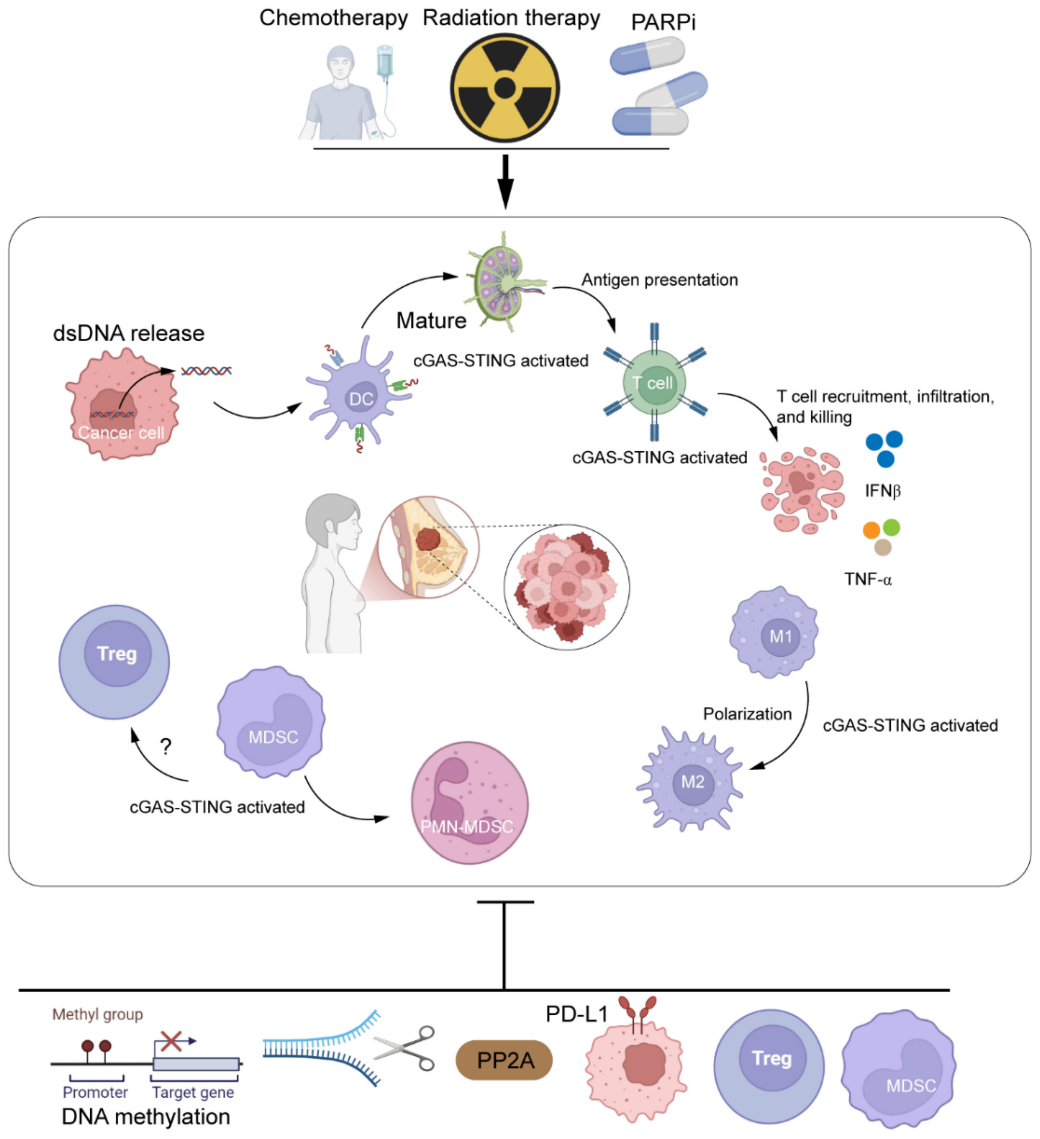
Role of cGAS-STING in breast cancer immune microenvironment regulation. Within breast tumor tissue (TNBC region), tumor cells exhibit dsDNA release (due to DNA damage/micronuclei) but often suppress STING via methylation or degradation. cGAS-STING activation in tumor-infiltrating DCs promotes maturation/antigen presentation, enhancing T-cell priming. Activated CD8+ T cells exert cytotoxic effects, while STING signaling drives TAM repolarization toward anti-tumor M1 phenotypes. Secreted IFN-β/TNF-α amplify immune responses but are counterbalanced by immunosuppressive elements: Tregs, MDSCs, PD-L1 expression, and STING negative regulators (phosphatases). Radiotherapy/chemotherapy induces dsDNA, fueling pathway activation. Visual: Green/red arrows denote pathway promotion/suppression. TAM, tumor-associated macrophage; MDSC, myeloid-derived suppressor cell. Created by BioRender.com (YQ294GQM5T).

The pathway exerts context-dependent effects: Antitumor: When the IRF3/IFN-I axis predominates, STING activation in dendritic cells potently primes CD8^+^ T-cell responses (91), transforming immunologically “cold” tumors into “hot” microenvironments conducive to tumor cell clearance. Protumor: In contexts favoring NF-κB signaling, chronic STING activation induces cancer-associated fibroblasts (CAFs) via WNT5A paracrine signaling, enriching stem-like cell populations and conferring therapy resistance (92). This exemplifies how the same pathway, under different spatiotemporal conditions, can be co-opted to promote tumor progression. As depicted in **Figure 4**, the cGAS-STING pathway exerts a dual regulatory role in the breast cancer microenvironment: on one hand, it activates dendritic cells (DCs) and CD8^+^ T cells; on the other hand, its immunostimulatory effects can be counteracted by immunosuppressive cells such as Tregs and MDSCs.

### 6.3 Therapeutic opportunities and challenges

Combination approaches are essential to overcome limitations: ICIs: Counteract STING-induced PD-L1 upregulation in TNBC. PARP inhibitors: Synergize with STING agonists in BRCA1-deficient models (93).

Targeted delivery: Nanocarriers enhance intratumoral agonist concentration. Dual-pathway blockade addresses pro-metastatic signaling: IL-6/STAT3 axis inhibition, reverses cGAS-STING-driven survival signals. Anti-IL-6R [tocilizumab (94)], suppresses inflammation-mediated metastasis in preclinical models.

### 6.4 Subtype-specific immunotherapeutic implications of cGAS-STING

The cGAS-STING signaling pathway exhibits distinct regulatory patterns and therapeutic implications across breast cancer subtypes. In BRCA1-deficient TNBC, PARP inhibitor olaparib activates the cGAS/STING pathway in tumor cells, leading to dendritic cell-dependent CD8+ T cell recruitment and antitumor immunity, with more pronounced effects in HR-deficient models and demonstrated STING pathway dependency (95). This suppression creates therapeutic opportunities for STING agonists. In endocrine-resistant ER+HER2- breast cancer, while hyperactivated AKT1 suppresses cGAS-STING signaling by binding to TBK1 and disrupting the TBK1/STING/IRF3 complex, combining AKT1 inhibitors with STING agonists restores innate immune activation and impairs tumor growth in preclinical models (96). In Herceptin-resistant HER2+ breast cancer, an immune-related prognostic index (IRPI) identifies suppressed type I interferon and cGAS-STING signaling as key mediators of immune escape, while combining STING agonists with the HER2-targeted ADC DS-8201 presents a promising strategy to overcome therapeutic resistance (97).

## 7 Clinical translation: from preclinical to clinical

### 7.1 STING agonists in clinical development

Although no cGAS/STING-targeted therapies have received FDA or EMA approval for cancers, including breast cancer, multiple STING agonists are under clinical evaluation and can be categorized into three classes:

Natural Cyclic Dinucleotides (CDNs): c-diGMP, c-diAMP, 2’3’-cGAMP: Exhibit potent antitumor activity in preclinical models (melanoma, colon cancer) but face ENPP1-mediated extracellular hydrolysis and poor biodistribution, limiting clinical advancement (98).

Synthetic CDN Agonists: ADU-S100 (MIW815): Mechanism: Phosphorothioate modifications enhance stability to trigger STING-TBK1-IRF3 signaling. Clinical Status: A phase Ib study demonstrated that the combination of the STING agonist MIW815 and the PD-1 antibody spartalizumab was well tolerated (maximum tolerated dose not reached) in patients with advanced solid tumors/lymphomas, including those with anti-PD-1 refractory disease; however, antitumor activity was limited (ORR 10.4%). Common adverse events included pyrexia (22%) and injection site pain (20%), while pharmacodynamic biomarkers confirmed target engagement (99). MK-1454: Mechanism: STING/PD-1 synergy overcomes resistance via intratumoral cytokine activation. Clinical Status: STING/PD-1 synergy overcomes resistance via intratumoral cytokine activation. Clinical Status: In a Phase I trial (NCT03010176), the combination of intratumoral MK-1454 with pembrolizumab elicited a compelling systemic anti-tumor response. Notably, select patients with advanced solid tumors experienced regression not only in the injected lesions but also in distant, non-injected metastases. This observation provides tangible clinical evidence that STING agonism can ignite a systemic immune response capable of targeting established tumors throughout the body. To date, MK-1454 remains under investigation in early-phase clinical trials, and its progression into later-phase studies is awaited to further define its therapeutic profile and efficacy in larger patient cohorts, including those with breast cancer. This strategy is strongly supported for further evaluation in immunotherapy-resistant cancers (100). E7766: Mechanism: Macrocyclic structure optimizes STING engagement. Preclinical Data: Induced durable immune memory in TNBC and bladder cancer models. Non-nucleotide Small Molecules

Non-nucleotide Small Molecules: diABZI: Intravenous administration of the novel small-molecule STING agonist diABZI (a linked amidobenzimidazole derivative) elicited strong anti-tumor activity in immunocompetent mice with established syngeneic colon tumors, resulting in complete and lasting regression of tumors (101). MSA-2: MSA-2, the first oral STING agonist, synergized with the anti-TGF-β/PD-L1 bispecific antibody YM101 to remarkedly retard tumor growth in both immune-excluded and immune-desert murine models. This combination simultaneously boosted innate/adaptive immunity and overcame immunotherapy resistance, demonstrating superior efficacy over monotherapies (102).

### 7.2 Breast cancer-focused clinical trials

Early Clinical Insights: Efficacy Signals: Eribulin uniquely enhances STING agonist-induced IFNβ expression and IRF3 phosphorylation in TNBC models and immune cells via potentiation of RNA-sensing pathways downstream of microtubule disruption, functioning as an immune adjuvant that synergizes with STING agonists to improve antitumor efficacy *in vivo*, supporting its combinatorial use with immunotherapy (105); The novel, structurally complex STING agonist MK-1454, discovered through rational design and optimization, demonstrated potent intratumoral cytokine induction and antitumor activity in mouse models; its efficacy, particularly in anti-PD-1 resistant tumors, was significantly enhanced when combined with a PD-1 antibody, supporting its clinical development in combination with pembrolizumab for immunotherapy-resistant cancers (100). Safety: Grade 1–2 injection-site reactions and transient lymphopenia dominate; dose-dependent IL-6 surges require vigilant monitoring. Biomarker Gaps: STING promoter methylation and ENPP1 overexpression predict resistance—demanding biomarker-driven patient selection.

These efforts are complemented by early-phase trials evaluating novel platforms such as oncolytic viruses and nanoparticle-based delivery systems to achieve more potent and targeted STING activation.

### 7.3 Approved therapies with indirect pathway modulation

Immune Checkpoint Inhibitors (ICIs): Pembrolizumab: Pembrolizumab-chemotherapy demonstrated significantly improved progression-free survival (PFS) in PD-L1-enriched metastatic TNBC, with median PFS of 9.7 months *vs*. 5.6 months (HR 0.65, 95% CI 0.49–0.86; P = 0.0012) in patients with CPS ≥10. The treatment effect increased with PD-L1 enrichment, though efficacy was not significant in the CPS ≥1 population [KEYNOTE-355] (15). Radiotherapy: Induces micronuclei-derived cytosolic DNA, activating cGAS-STING-dependent abscopal effects; clinically enhances systemic responses in oligometastatic breast cancer. PARP Inhibitors & Chemotherapy: Olaparib: Final analysis of the Phase III OlympiAD trial showed no statistically significant overall survival benefit for olaparib versus chemotherapy (TPC) in patients with germline BRCA-mutated, HER2-negative metastatic breast cancer (median OS 19.3 *vs* 17.1 months; HR 0.90, p=0.513), though a potential OS benefit was observed in patients not previously treated with chemotherapy for metastatic disease (first-line HR 0.51); olaparib demonstrated a manageable safety profile with low discontinuation rates and no cumulative toxicity (106). Anthracyclines: Promote tumor-derived DNA release into TME, amplifying DC cross-priming via cGAS.

In mouse models of breast cancer metastasis, evidence reveals that irradiated mesenchymal stromal cells exhibit activated cGAS-STING signaling, demonstrated by upregulated type I interferon-related genes and CCL5 expression, which promotes lung metastasis in a macrophage-dependent manner (79). However, this persistent signaling is associated with T-cell exhaustion and an immunosuppressive TME, rather than productive immunity. A key resistance mechanism involves tumor-derived exosomes carrying ENPP1, which hydrolyzes extracellular cGAMP - including LL-37-bound cGAMP - thereby suppressing STING activation in immune cells and inhibiting T cell infiltration. This creates a paradoxical scenario where the pathway is actively suppressed through extracellular degradation of its messenger (107).

## 8 Challenges and future perspectives

### 8.1 Fundamental scientific barriers to clinical translation

The cGAS-STING pathway exhibits a dual role: while it drives antitumor immunity, its non-canonical activation (NF-κB) can induce immunosuppressive molecules such as PD-L1 and IDO, thereby promoting immune evasion, as seen in melanoma models. In breast cancer, Chromosomal Instability (CIN)-activated cGAS-STING-IL-6 signaling enhances tumor cell survival via STAT3 (10), fostering a pro-metastatic microenvironment. Additionally, sustained STING activation in senescent cells triggers the Senescence-Associated Secretory Phenotype (SASP), whose chronic inflammation may fuel therapy resistance and metastasis. Breast Cancer Case Example: In TNBC, STING-dependent IL-6 secretion maintains cancer stemness through STAT3 signaling, countering therapeutic responses.

Immunosuppressive Cells: STING activation induces IL-35 secretion by B cells (108), suppressing NK cell density. Subtype Variability: Melanomas often silence the pathway via cGAS/STING promoter hypermethylation, whereas TNBC shows paradoxical pathway hyperactivation (driven by CIN) that promotes protumorigenic factors.

No reliable biomarkers exist to identify patients responsive to STING agonists. STING-deficient tumors exhibit heightened sensitivity to oncolytic viruses, yet intact cGAS-STING signaling may suppress viral replication via antiviral responses, complicating therapeutic stratification.

Clinical trials of STING agonists in advanced solid tumors, including breast cancer, reveal nuanced response patterns. While single-agent activity has been limited, combination approaches show promise. Patients with low-risk tumors characterized by Tertiary Lymphoid Structures (TLS) may benefit from immune checkpoint inhibitors regardless of PD-L1 status, whereas high-risk patients with elevated tumor mutational burden show better responses to chemotherapy (109). However, systemic administration is often limited by cytokine release syndrome (CRS), underscoring the need for improved delivery platforms to achieve therapeutic intratumoral concentrations without systemic toxicity.

### 8.2 Technical hurdles in drug development and combination therapy

Natural CDN Drawbacks: Endogenous cGAMP is rapidly hydrolyzed by ENPP1, and its hydrophilicity impedes cellular uptake. Solutions: Synthetic Agonists: This study synthesized oxaliplatin(IV) prodrugs conjugated with the STING agonist MSA-2; while albumin-targeted derivatives enhanced platinum tumor accumulation and reduced oxaliplatin-associated hematotoxicity, *in vivo* and *in vitro* data revealed no synergistic antitumor effect between simultaneously released oxaliplatin and MSA-2 (efficacy comparable to monotherapies), challenging the therapeutic potential of such dual-release strategies but confirming platinum(IV) carriers improve pharmacokinetic properties (110). Targeted Delivery Systems: Lipid nanoparticles (LNPs) were employed as a delivery platform with the potential to facilitate nucleic acid delivery. This study focused on evaluating the sustained expression profile and safety of the LNP-formulated pDNA (111).

Combining STING agonists with immune checkpoint inhibitors (ICIs) may induce systemic inflammation (cytokine release syndrome). Optimization Strategies: Dose Titration: Low-dose CAR-T cells combined with STING agonists enhance TME activation and efficacy in renal carcinoma. Temporal Sequencing: Radiotherapy-induced STING activation upregulates PD-L1, necessitating careful scheduling with anti-PD-1 agents to maximize synergy.

### 8.3 Strategic roadmap for future research and clinical integration

Metabolic Reprogramming: Investigate how mitochondrial damage releases mtDNA, activating cGAS-STING and immune checkpoints to drive tumor progression. Microbiome Modulation: Explore gut microbiota-STING interactions in breast cancer immunity, an area extending beyond the current literature.

Suppressing Negative Regulators: Develop inhibitors against ENPP1 to preserve cGAMP; explore targeting viral proteins (HPV18 E7) that inhibit STING-NF-κB signaling. Exploiting Synergistic Targets: Evaluate PARP inhibitors for their role in amplifying cGAS-STING to enhance CAR-T efficacy; investigate Manganese ions (Mn²^+^) as potentiators of STING-agonist-induced anti-PD-1 responses.

Counteracting TNBC Protumorigenesis: The cGAS-STING pathway promotes tumorigenesis by enabling IL-6/STAT3-dependent survival of chromosomally unstable (CIN) cancer cells, revealing a targetable vulnerability exploitable by IL-6R blockade (tocilizumab) and explaining why this pathway is rarely inactivated in primary tumors (10). Blocking Metastatic Cascade: Develop strategies to target cGAMP transfer between tumor cells and astrocytes to suppress brain metastasis. Overcoming Resistance: HRD activates cGAS-STING-IFN signaling to sensitize tumors to PARP inhibitors (PARPi), but resistance suppresses this pathway. Combining PARPi + STING agonist overcomes resistance via NK cell-dependent immunity (112).

To bridge the gap between mechanistic understanding and clinical application, we propose the following concrete strategies: Biomarker-Driven Patient Selection: Proposal: We advocate for the routine pathological assessment of STING and ENPP1 expression in diagnostic TNBC biopsies. This should be validated as a standard practice to guide patient selection for upcoming clinical trials, ensuring that individuals most likely to benefit from STING-targeted therapies are appropriately enrolled. Defining Novel Trial Endpoints: Proposal: Beyond traditional RECIST criteria, clinical trials should incorporate STING-induced Tertiary Lymphoid Structure (TLS) formation as a surrogate endpoint for productive immune activation. This would provide an early and mechanistically informed indicator of treatment efficacy. Optimizing Therapeutic Sequencing and Delivery: Proposal: Preclinical data suggest that the timing of STING agonist administration relative to other modalities (radiotherapy, ICIs) is critical. Clinical trials must systematically evaluate different sequencing schedules to identify regimens that maximize synergy and minimize toxicity.

## 9 Conclusion

The cGAS-STING signaling pathway stands as a central orchestrator of innate and adaptive antitumor immunity within the breast cancer microenvironment. Its ability to detect tumor-derived cytosolic DNA—such as that arising from chromosomal instability (CIN) prevalent in TNBC—triggers a potent immune cascade. This cascade involves type I interferon (IFN-I) production, dendritic cell (DC) maturation, and CD8^+^ T cell priming and recruitment, collectively positioning cGAS-STING as a molecular rheostat capable of converting immunologically “cold” tumors into “hot,” inflamed microenvironments.

Leveraging this pathway holds immense promise both as a therapeutic target and as a cornerstone for innovative combination strategies. Preclinical and emerging clinical evidence strongly supports the synergistic enhancement of immune checkpoint inhibitor (ICI) efficacy when combined with STING agonists. Furthermore, rational combinations with DNA-damaging agents like PARP inhibitors or radiotherapy exploit endogenous pathway activation, while targeting downstream protumorigenic outputs counters the pathway’s paradoxical roles.

However, the translation of cGAS-STING targeting from bench to bedside faces significant hurdles. These include: Toxicity Concerns: Systemic activation risks cytokine release syndrome (CRS) and inflammatory toxicities driven by IFN-I and IL-6, necessitating optimized dosing and delivery strategies. Delivery Challenges: The inherent instability of natural cyclic dinucleotides (CDNs) like cGAMP due to ENPP1 hydrolysis, combined with poor bioavailability and cellular uptake, demands advanced delivery platforms such as lipid nanoparticles (LNPs), polymeric carriers, or orally bioavailable small molecules. Efficacy Prediction: Identifying responders remains difficult due to pathway heterogeneity and a lack of robust biomarkers. Looking forward, realizing the transformative potential of cGAS-STING pathway modulation in breast cancer immunotherapy requires focused efforts: Next-Generation Agonists & Delivery: Develop tumor-selective agonists (protease-activated prodrugs) and refine delivery systems (nanocarriers, localized administration) to maximize intratumoral efficacy while minimizing systemic toxicity. Precision Combination Strategies: Design mechanism-driven combinations informed by tumor biology. This includes rational pairing with ICIs, PARP inhibitors, metabolic modulators, or therapies targeting CAFs/TAMs to counteract immunosuppression and protumorigenic outputs like IL-6 or PD-L1. Blocking cGAMP transfer in metastatic niches (brain) is also critical. Advanced Biomarkers and Actionable Trial Designs: Moving beyond PD-L1 through integrated multi-omics approaches. To bridge the translational gap, future clinical trials should adopt biomarker-driven designs. We recommend that Phase II trials of STING-targeted therapies prospectively stratify TNBC patients based on composite biomarkers, such as CIN scores and STING/ENPP1 protein expression assessed by standardized IHC. Furthermore, validating STING-induced Tertiary Lymphoid Structure (TLS) formation as a surrogate endpoint, beyond traditional RECIST criteria, could provide an early and mechanistically informed indicator of productive immune activation.

**Figure 5:**
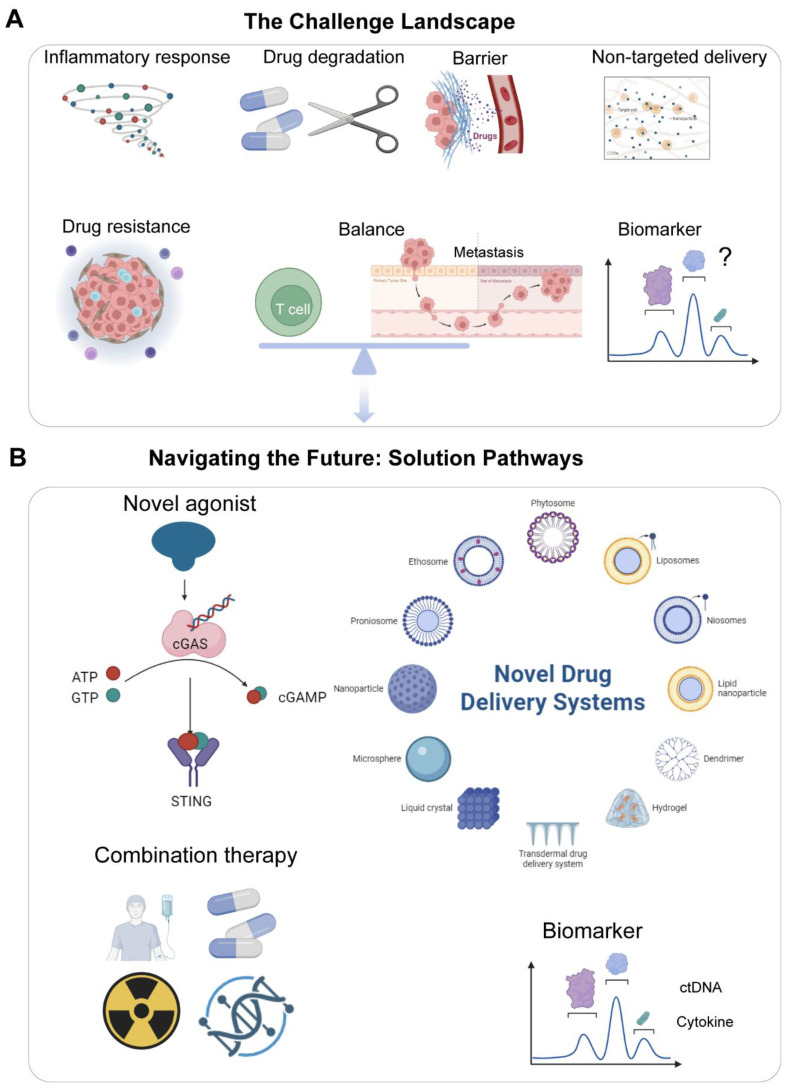
Challenges and future directions in cGAS-STING-targeted breast cancer therapy. **(A)** The Challenge Landscape: Systemic toxicity/cytokine release; poor drug stability/bioavailability; tumor-intrinsic resistance (STING silencing); paradoxical pro-metastatic effects; lack of predictive biomarkers. **(B)** Navigating the Future: Solution Pathways: (1) Next-generation agonists with improved stability/tumor selectivity; (2) Advanced delivery systems (stimuli-responsive nanoparticles, local implants); (3) Rational combinations (with ICI, PARPi, epigenetic modulators); (4) Targeting negative regulators (PP2A); (5) Biomarker discovery (tissue/blood-based) for patient stratification; (6) Deciphering context-dependent roles in metastasis/metabolism/microbiome interactions. Visual: Warning icons (challenges) linked to solution pathways (arrow networks). ICI, immune checkpoint inhibitor. Created by BioRender.com (YQ294GQM5T).

By addressing these challenges through continued innovation in drug design, delivery technologies, and biomarker-guided patient selection, targeted manipulation of the cGAS-STING pathway offers a compelling avenue to significantly improve outcomes, particularly for patients with aggressive and immunotherapy-resistant breast cancer subtypes like TNBC. Successfully harnessing this pathway has the potential to shift the treatment paradigm towards achieving more durable remissions. As summarized in **Figure 5**, current cGAS-STING-targeted therapies still face challenges including toxicity, suboptimal delivery efficiency, and a lack of reliable biomarkers. Future efforts should focus on developing next-generation agonists, precision combination strategies, and novel biomarkers to advance clinical translation.
